# CBX7 and HMGA1b proteins act in opposite way on the regulation of the *SPP1* gene expression

**DOI:** 10.18632/oncotarget.2777

**Published:** 2015-01-20

**Authors:** Romina Sepe, Umberto Formisano, Antonella Federico, Floriana Forzati, André Uchimura Bastos, Daniela D'Angelo, Nunzio Antonio Cacciola, Alfredo Fusco, Pierlorenzo Pallante

**Affiliations:** ^1^ Istituto per l'Endocrinologia e l'Oncologia Sperimentale (IEOS) “G. Salvatore”, Consiglio Nazionale delle Ricerche (CNR), c/o Dipartimento di Medicina Molecolare e Biotecnologie Mediche (DMMBM), Università degli Studi di Napoli “Federico II”, 80131 Naples, Italy; ^2^ Laboratório as Bases Genéticas dos Tumores da Tiroide, Disciplina de Genética, Universidade Federal de São Paulo – UNIFESP, Rua Pedro de Toledo 669, 11° andar, 04039-032 São Paulo, SP, Brazil; ^3^ Instituto Nacional de Câncer - INCA, Praça da Cruz Vermelha 23, Centro, 20230-130 Rio de Janeiro, RJ, Brazil

**Keywords:** Osteopontin, CBX7, HMGA1b, NF-κB, thyroid carcinomas

## Abstract

Several recent studies have reported the Polycomb Repressive Complex 1 member CBX7 as a tumor-suppressor gene whose expression progressively decreases in different human carcinomas in relation with tumor grade, malignant stage and poor prognosis. We have previously demonstrated that CBX7 is able to inhibit the expression of the *SPP1* gene, encoding the chemokine osteopontin that is over-expressed in cancer and has a critical role in cancer progression.

Here, we have analyzed the mechanism by which CBX7 regulates the *SPP1* gene expression. We show that the *SPP1* transcriptional regulation mechanism involves the CBX7-interacting protein HMGA1b, that acts as a positive regulator of the *SPP1* gene. In fact, we demonstrate that, in contrast with the transcriptional activity of CBX7, HMGA1b is able to increase the *SPP1* expression by inducing the activity of its promoter. Moreover, we show that CBX7 interferes with HMGA1b on the *SPP1* promoter and counteracts the positive transcriptional activity of HMGA1b on the *SPP1* expression.

Furthermore, since we found that also the NF-κB complex resulted involved in the modulation of the *SPP1* expression in thyroid cells, we suppose that CBX7/HMGA1b/NF-κB could take part in the same transcriptional mechanism that finally leads to the regulation of the *SPP1* gene expression.

Taken together, our data show the important role played by CBX7 in the negative regulation of the *SPP1* gene expression, thus contributing to prevent the acquisition of a malignant phenotype.

## INTRODUCTION

The chromobox homolog 7 (CBX7) protein is a member of the Polycomb Repressive Complex (PRC) 1, a macromolecular complex that, together with the PRC2, contributes to silence genes involved in cell development and differentiation [1, 2]. In particular, CBX7 exerts its role of gene transcriptional regulator by binding specific histone modification sites on the chromatin structure thus modulating the expression of specific genes by interacting with other PRC1/2 members [3, 4].

The role of CBX7 in carcinogenesis is controversial and not completely defined so far. CBX7 over-expression is associated with poor prognosis in ovarian adenocarcinomas through the inhibition of the TRAIL-induced apoptotic pathway [5] and its expression leads to the expansion of cellular lifespan in human prostate primary epithelial cells [6] and in mouse embryonic fibroblasts (MEFs) through the repression of the *Ink4a/Arf* locus [7]. Moreover, the over-expression of Cbx7 in the lymphoid compartments of mouse model enhances T-cell lymphomagenesis and induces highly aggressive B cell lymphomas *in vivo* through a mechanism that involves the cooperation with c-Myc protein [8].

However, in several human carcinomas CBX7 acts as a tumor suppressor gene preventing the acquisition of a malignant phenotype. In fact, CBX7 resulted progressively down-regulated in a wide array of human carcinomas including thyroid [9], colon [10], breast [11], pancreas [12] and lung [13] carcinomas in relation to tumor grade and malignant stage. A strong association between the loss of CBX7 expression and a reduced survival was also observed in patients affected by pancreatic [12] and colon [10] carcinomas. CBX7 is able to negatively regulate cell proliferation in thyroid [9], colon [10] and lung [13] carcinoma cell lines in which the expression of CBX7 was restored while MEFs from *Cbx7^−/−^* mice showed a higher cell proliferation rate compared with wild type ones [13]. Moreover, mice knock-out for *Cbx7* gene had a higher incidence of lung and liver carcinomas than heterozygous and wild type mice [13], suggesting the anti-oncogenic role played by the CBX7 protein in carcinogenesis.

By interacting with several proteins, CBX7 positively or negatively modulates the expression of genes involved in different biological processes. CBX7 contributes, in fact, to prevent cancer progression by positively regulating the expression of the E-cadherin through the interaction with the HDAC2 protein and the following inhibition of the HDAC2 activity [14]. Moreover, CBX7 is involved in the cell cycle regulation by negatively regulating the cyclin E expression counteracting the transcriptional activity of HMGA1b [13], a non-histone chromatin protein strongly expressed in several human carcinomas [15] that plays a pivotal role in cancer progression [16, 17] and in control of cell fate [18]. Furthermore, a recent study demonstrates that the loss of CBX7 observed in thyroid carcinomas leads to a negative or positive regulation of key genes involved in tumorigenesis, as the AP1 complex members *FOS* and *FOSb*, and the chemokine *SPP1*, respectively [19].

In order to investigate the role of CBX7 in cancer and the mechanism accounting for the association between the loss of the CBX7 expression and a high malignant grade and poor prognosis, we decided to characterize the molecular mechanism by which CBX7 regulates the expression of its modulated-gene *SPP1*, encoding the osteopontin protein well known to be involved in tumor cell migration and invasion and whose over-expression is associated with high malignant phenotype [20, 21] and the presence of tumor metastases [22]. Since recently it has been demonstrated that HMGA1b is an interacting protein of CBX7 [13], the aim of this study is to investigate the involvement of the CBX7/HMGA1b complex in the transcriptional regulation of the *SPP1* gene. Here, we show that CBX7 and HMGA1b act in opposite way on the *SPP1* gene expression and that CBX7 is able to counteract the positive transcriptional effect of HMGA1b on the *SPP1* expression. Finally, we demonstrate that this transcriptional mechanism could involve also the NF-κB complex, a positive regulator of *SPP1* [23].

In conclusion, the loss of CBX7 expression associated to HMGA1b over-expression contributes to the positive regulation of the *SPP1* gene expression in cancer.

## RESULTS

### HMGA1b protein positively regulates the expression of the *SPP1* gene

It has been recently demonstrated that the CBX7 protein inhibits the expression of the *SPP1* gene [19]. Moreover, we have reported that the non-histone chromatin protein HMGA1b interacts with CBX7 [13] and that the CBX7/HMGA1b complex is involved in the modulation of cyclin E expression. Therefore, we asked whether the interaction between these two proteins could take part also in the regulation of the *SPP1* gene.

**Figure 1:**
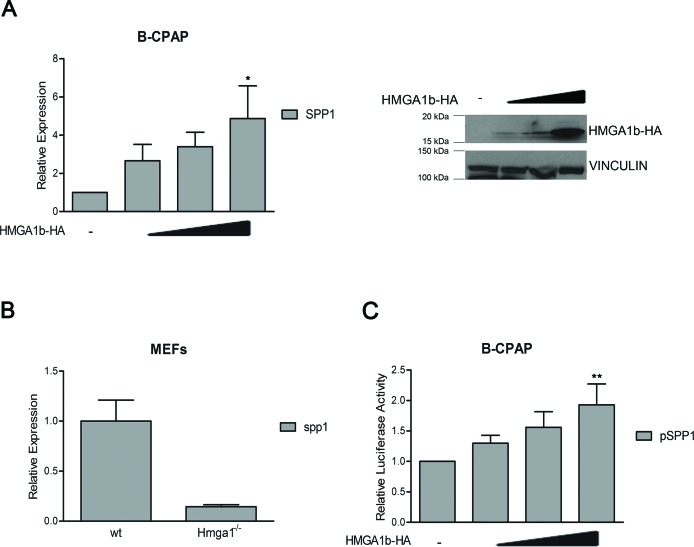
HMGA1b induces the expression of the *SPP1* gene **(A)** Left Panel. qRT-PCR analysis of *SPP1* in B-CPAP cells transfected with increasing amount (1 μg, 3 μg and 5 μg) of HMGA1b-HA expressing vector. The total amount of the transfected DNA was balanced with the empty vector. Relative Expression represents values normalized to control cells transfected with the only empty vector, set equal to 1. Values are the mean of four independent experiments performed in triplicate ± SEM. Kruskal-Wallis test followed by Dunn's post test: *p < 0.05 compared to control cells. Right Panel. Immunoblot analysis showing increasing expression of HMGA1b-HA. Vinculin was used as control to normalize the amount of protein loaded. **(B)** qRT-PCR analysis of *Spp1* expression in mouse embryonic fibroblasts (MEFs) from *Hmga1^−/−^* mice compared to wild type (wt) MEFs, set equal to 1. qRT-PCR analysis was performed in duplicate and reported values represent the mean ± SEM. **(C)** Luciferase assays performed in B-CPAP cells transfected with the *SPP1-luc* vector. The total amount of the transfected DNA was balanced with the empty vector. Relative Luciferase Activity (pSPP1) was compared to that observed in cells transfected with the only empty vector, set equal to 1. Values are the mean of five independent experiments performed at least in duplicate ± SEM. Kruskal-Wallis test followed by Dunn's post test: **p < 0.01 compared to control cells.

To this aim, we first evaluated whether HMGA1b was able to regulate the *SPP1* expression by transiently transfecting the papillary thyroid carcinoma cell line B-CPAP, that shows low expression of the *SPP1* gene [24], with increasing amount of a vector expressing the HMGA1b protein fused with the HA epitope (HMGA1b-HA). Quantitative RT-PCR (qRT-PCR) analysis showed that HMGA1b protein increased the expression levels of the *SPP1* gene in relation to the amount of the HMGA1b protein (Figure 1A). Consistently, we found that MEFs obtained from mice knock-out for the *Hmga1* gene had a lower expression of *Spp1* compared to wild type MEFs (Figure 1B). Thereafter, to evaluate the effect of HMGA1b on the activity of the *SPP1* promoter, we transiently transfected B-CPAP cells, showing low activity of the *SPP1* promoter (Supplementary Figure 1), both with a vector encoding the *luciferase* gene under the control of 1000 bp region upstream the TSS of the *SPP1* promoter and increasing amount of a vector encoding HMGA1b-HA protein. As shown in Figure 1C, HMGA1b protein was able to enhance the *SPP1* promoter activity in a dose-dependent manner suggesting that HMGA1b is a positive transcriptional regulator of the *SPP1* gene. In addition, qRT-PCR assay performed on a panel of human thyroid carcinomas of different histotypes showed a positive correlation between *HMGA1b* and *SPP1* expression while a negative correlation was observed between *CBX7* and the expression of *HMGA1b* and *SPP1* (Supplementary Figure 2).

### CBX7 and HMGA1b bind the *SPP1* promoter *in vivo*

**Figure 2:**
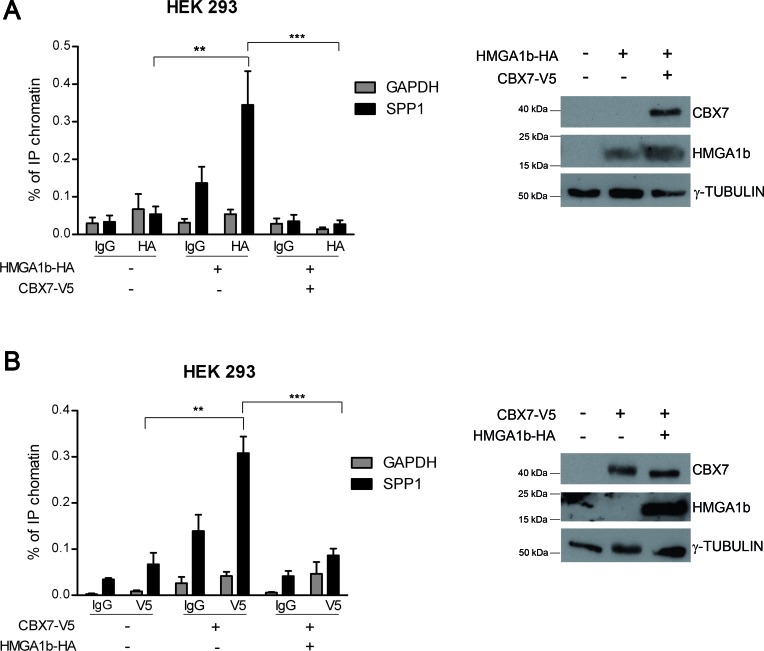
CBX7 and HMGA1b bind the *SPP1* promoter *in vivo* HEK 293 cells were transiently transfected with a vector expressing HMGA1b-HA or CBX7-V5 or with the only empty vector. The chromatin was immunoprecipitated (IP) by using antibodies against HA (**A**, left panel) or V5 (**B**, left panel) tag. IgG were used as negative control. The IP chromatin was analyzed by qRT-PCR assay performed in triplicate with primers specific for the *SPP1* promoter. *GAPDH* promoter primers were used as control of the binding specificity. Values are the mean of three **(A)** or two **(B)** independent experiments ± SEM. Mann Whitney test: **p < 0.01 and ***p < 0.001. The right panel of the figure displays the immunoblot analysis confirming the expression of HMGA1b-HA and CBX7-V5. γ-Tubulin was used as control to normalize the amount of protein loaded.

Our results support the hypothesis that the deregulation of the CBX7/HMGA1b axis might contribute to cancer progression through the modulation of the *SPP1* gene expression. To test this hypothesis we performed chromatin immunoprecipitation (ChIP) assays in HEK 293 cells transiently transfected with a vector encoding the V5-tagged CBX7 (CBX7-V5) protein or the HMGA1b-HA protein or both vectors. The DNA-protein complexes were fixed and then immunoprecipitated by using antibodies against the HA or the V5 epitope. Aspecific IgG rabbit antibodies were used as a negative control of the experiment. The chromatin was then released by the immunocomplexes and analyzed by qRT-PCR assay using specific primers for the human *SPP1* promoter. As shown in Figure 2A, in presence of the HA antibodies we found that the amount of immunoprecipitated (IP) chromatin was higher in cells transfected with HMGA1b-HA vector than in those transfected with the empty vector. Interestingly, by this approach we observed that when both CBX7-V5 and HMGA1b-HA were expressed the amount of IP chromatin was lower than in presence of the only HMGA1b-HA protein, demonstrating that CBX7 displaces HMGA1b from the *SPP1* promoter. Moreover, the same result was obtained by performing ChIP assays with V5-antibodies. In fact, by these experiments, we confirmed that CBX7 binds the *SPP1* promoter, as already shown [19], and also found that when both CBX7 and HMGA1b were expressed the amount of IP chromatin was lower than in presence of the only CBX7 protein, suggesting that CBX7 and HMGA1b proteins counteract each other on the *SPP1* promoter *in vivo* (Figure 2B). No amplification was observed by using specific primers for the *GAPDH* promoter, indicating that the CBX7/HMGA1b binding is specific for the *SPP1* promoter.

### CBX7 counteracts the positive transcriptional effect of HMGA1b on the *SPP1* expression

**Figure 3:**
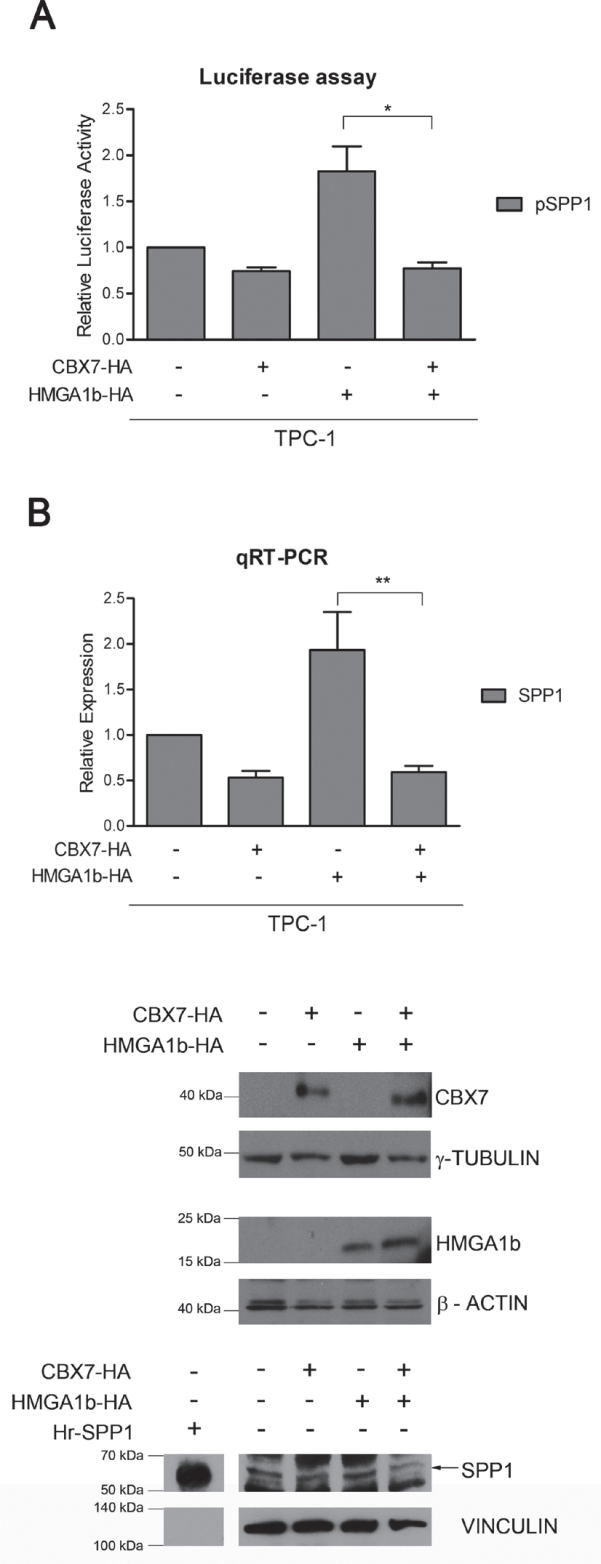
CBX7 counteracts the transcriptional effect of HMGA1b on the *SPP1* gene **(A)** Luciferase assays performed in TPC-1 cells transfected with the *SPP1-luc* vector and with single or both vectors expressing CBX7-HA or HMGA1b-HA. The total amount of the transfected DNA was balanced with the empty vector. Relative Luciferase Activity (pSPP1) was compared to that observed in cells transfected with the only empty vector, assuming that the control is equal to 1. Values are the mean of four independent experiments performed at least in duplicate ± SEM. Kruskal-Wallis test followed by Dunn's post test: *p < 0.05. **(B)** SPP1 expression analysis in TPC-1 cells transfected with single or both vectors expressing CBX7-HA or HMGA1b-HA. The total amount of the transfected DNA was balanced with the empty vector. Upper Panel. qRT-PCR analysis. Relative Expression represents values normalized to control cells transfected with the only empty vector, set equal to 1. Values are the mean of five independent experiments performed at least in duplicate ± SEM. Kruskal-Wallis test followed by Dunn's post test: **p < 0.01. Lower Panel. Immunoblot analysis confirming the expression of CBX7-HA, HMGA1b-HA and osteopontin (indicated by the black arrow). Human full length osteopontin recombinant protein (Hr-SPP1) was used as positive control. The recombinant protein signal was obtained from a short exposure of the same membrane. Proteins from the same experiment were loaded on three different gels. γ-Tubulin, β-Actin and Vinculin were used as control to normalize the amount of protein loaded.

Next, we analyzed the effect of the CBX7/HMGA1b complex on the modulation of the *SPP1* promoter activity. To this aim, we performed luciferase assays in the papillary thyroid carcinoma cell line TPC-1, showing high activity levels of the *SPP1* promoter (Supplementary Figure 1), transfected with a vector encoding the *luciferase* gene under the control of the *SPP1* promoter and with single or both vectors encoding the HA-tagged CBX7 (CBX7-HA) or the HMGA1b-HA protein. As shown in Figure 3A, we found that CBX7 and HMGA1b behaved in opposite way on the modulation of the *SPP1* promoter, but when both proteins were expressed, its activity showed low levels comparable to those observed in cells expressing the only CBX7-HA protein. Finally, we evaluated the transcriptional effects due to the simultaneous presence of CBX7 and HMGA1b proteins on the expression of the *SPP1* gene by transiently transfecting TPC-1 cells, expressing high levels of *SPP1* [24], with a vector encoding CBX7-HA or HMGA1b-HA or both vectors. By qRT-PCR and Western blot analysis, we observed that CBX7 was able to repress the *SPP1* expression while HMGA1b was able to induce it, as expected (Figure 3B). More interestingly, we found that when both proteins were expressed the *SPP1* expression decreased showing levels comparable to those observed in the presence of the only CBX7-HA protein. This suggests that the transcriptional repressive effect of CBX7 is stronger than the positive one exerted by HMGA1b (Figure 3B).

Taken together, these results demonstrate that CBX7 negatively regulates the expression of the *SPP1* gene by counteracting the activity of HMGA1b and that the presence of CBX7 makes HMGA1b protein unable to exert its role of positive regulator of the *SPP1* expression.

### CBX7 regulates cell migration through the block of HMGA1b and suppression of the *SPP1* gene expression

**Figure 4:**
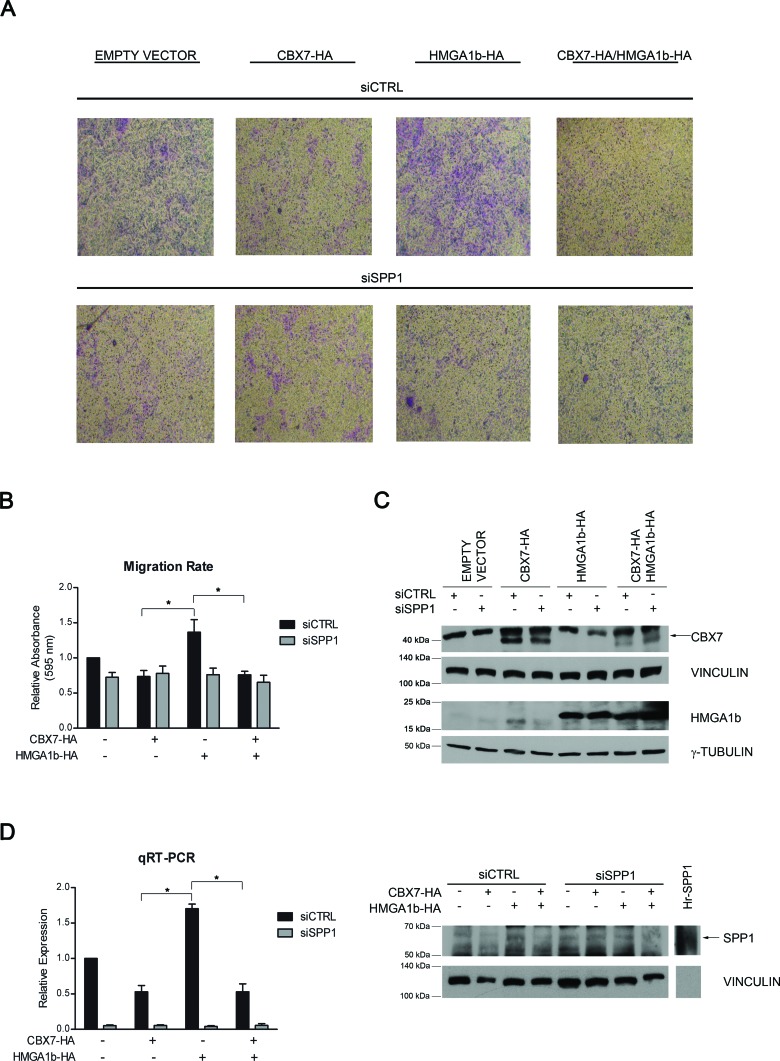
CBX7 and HMGA1b proteins modulate cell migration through the transcriptional regulation of the *SPP1* gene TPC-1 cells were co-transfected with vectors encoding CBX7-HA or HMGA1b-HA or with the only empty vector and with a specific siRNA for the *SPP1* mRNA or the control siRNA. The total amount of the transfected DNA was balanced with the empty vector. **(A)** Transwell pictures from a representative experiment. Magnification 50X. **(B)** To evaluate cell migration rate, transwells were de-stained and crystal violet solution was read at 595 nm. Values obtained were normalized to values of cells transfected with the only empty vector and the control siRNA (siCTRL), set equal to 1. Represented values are the mean of four independent experiments ± SEM. Kruskal-Wallis test followed by Dunn's post test: *p < 0.05. **(C)** Immunoblot analysis confirming the expression of CBX7-HA (indicated by the black arrow) and HMGA1b-HA proteins. Proteins from the same experiment were loaded on two different gels. γ-Tubulin and Vinculin were evaluated to normalize the amount of the protein used. **(D)** Left Panel. Relative Expression represents values normalized to control cells transfected with the only empty vector and the control siRNA (siCTRL), set equal to 1. Represented values are the mean of four independent experiments performed in triplicate ± SEM. Kruskal-Wallis test followed by Dunn's post test: *p < 0.05. Right Panel. Immunoblot analysis confirming the expression of osteopontin (indicated by the black arrow). Human full length osteopontin recombinant protein (Hr-SPP1), used as positive control, was separated from samples by the molecular weight marker (not shown). Vinculin was used as control to normalize the amount of protein loaded.

Osteopontin is a protein well known to be involved in cancer progression through the promotion of cell migration and invasion with the resulting formation of tumor metastases [22]. Therefore, we evaluated the functional effects exerted by CBX7 and HMGA1b on cell migration through the transcriptional regulation of the *SPP1* gene expression. To this aim, TPC-1 cells transfected with vectors encoding CBX7-HA or HMGA1b-HA or both vectors were treated with a siRNA specific for the *SPP1* mRNA or with the aspecific non silencing siRNA. 24 h after transfection, cells were seeded in a transwell and cell migration ability was evaluated after additional 24 h (Figure 4A). As shown in Figure 4B and 4C, cells expressing HMGA1b and treated with the aspecific control siRNA showed a higher migration rate than control cells transfected with the only empty vector and treated with the aspecific siRNA. On the contrary, cells expressing CBX7 or both CBX7 and HMGA1b and treated with the control siRNA showed a lower migration rate than cells expressing the only HMGA1b protein or transfected with the only empty vector, demonstrating that CBX7 is able to reduce cell migration rate counteracting the HMGA1b activity. Moreover, when the *SPP1* expression was silenced through a specific siRNA, the migration ability of cells expressing HMGA1b decreased, suggesting the involvement of the osteopontin in the migration of these cells. Conversely, cells treated with the *SPP1* siRNA and expressing CBX7 or both CBX7 and HMGA1b showed a low migration rate comparable to that of the same transfected cells treated with the control siRNA, suggesting that the CBX7 mediated reduction of cell migration involves the down-regulation of the *SPP1* gene expression. Osteopontin expression was evaluated by qRT-PCR and Western blot analysis (Figure 4D). Moreover, ELISA assay showed a strongly osteopontin reduction when transfected cells were treated with the specific *SPP1* siRNA (data not shown). In conclusion, all these data reinforce the hypothesis that the cell migration ability could be due to the regulation of the *SPP1* gene expression mediated by CBX7 and HMGA1b proteins.

### CBX7 and HMGA1b regulate the *SPP1* gene expression through the modulation of the NF-κB activity

**Figure 5:**
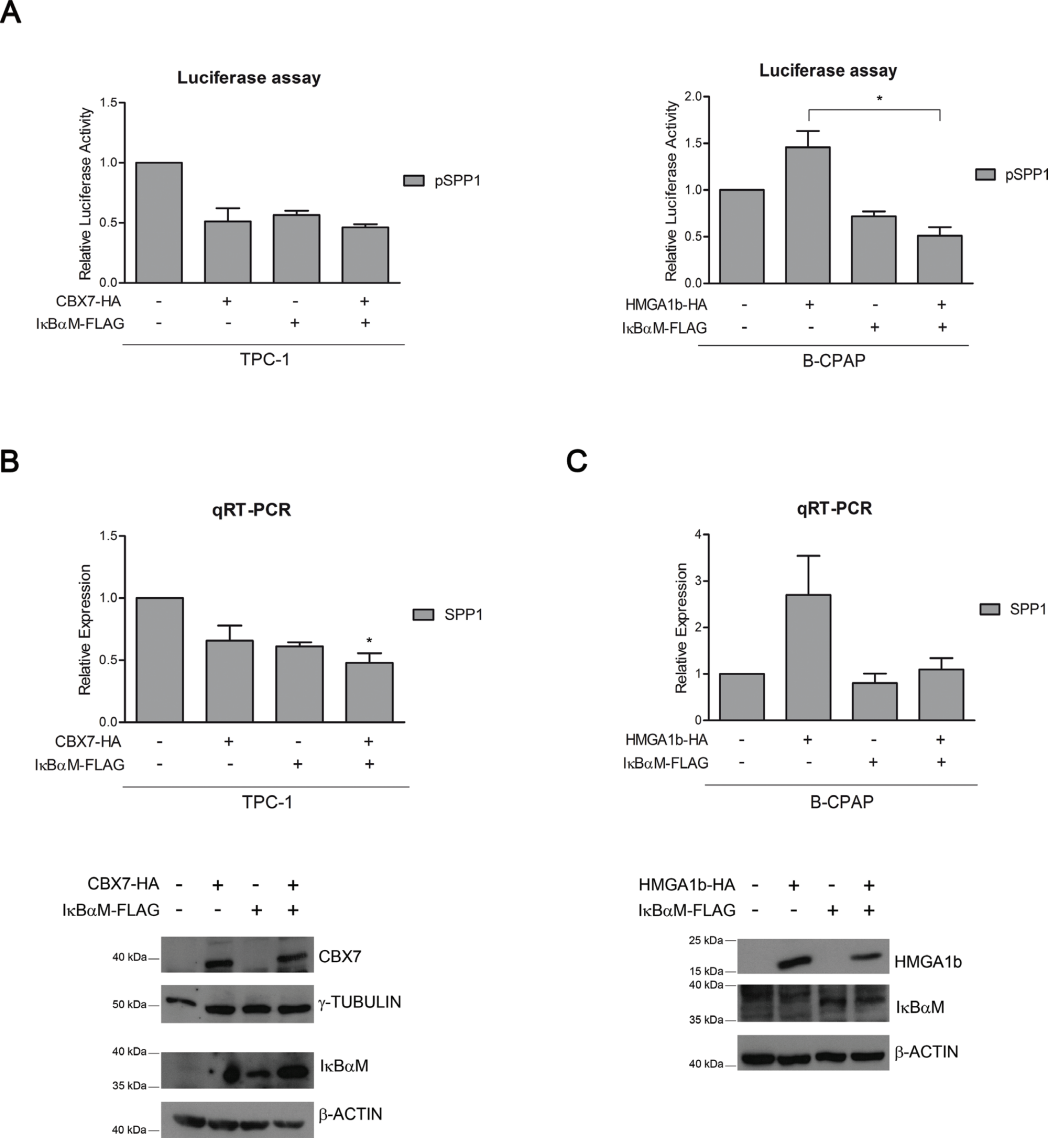
CBX7, HMGA1b and NF-κB are involved in the regulation of the *SPP1* gene expression **(A)** Luciferase assays performed in TPC-1 (left panel) and B-CPAP (right panel) cells transfected with the *SPP1-luc* vector and with CBX7-HA or HMGA1b-HA and the IκBαM-FLAG expressing vector. The total amount of the transfected DNA was balanced with the empty vector. Relative Luciferase Activity (pSPP1) was compared to that observed in cells transfected with the only empty vector, set equal to 1. Values are the mean of three independent experiments performed in duplicate in TPC-1 cells and at least in duplicate in B-CPAP cells ± SEM. B-CPAP, Kruskal-Wallis test followed by Dunn's post test: *p < 0.05. **(B, C)** Upper Panel. qRT-PCR analysis performed in TPC-1 and B-CPAP cells expressing IκBαM-FLAG and CBX7-HA or HMGA1b-HA. The total amount of the transfected DNA was balanced with the empty vector. Relative Expression represents values normalized to control cells transfected with the only empty vector, set equal to 1. Values are the mean of four independent experiments performed in triplicate. TPC-1, Kruskal-Wallis test followed by Dunn's post test: *p < 0.05 compared to control cells. Lower Panel. Immunoblot analysis confirming the expression of CBX7-HA, HMGA1b-HA and IκBαM-FLAG. For TPC-1 cells **(B)**, proteins from the same experiment were loaded on two different gels. γ-Tubulin and β-Actin were used as control to normalize the amount of protein loaded.

A recent study has demonstrated that the activation of the NF-κB complex is responsible of the over-expression and secretion of the osteopontin protein [23]. Since it has been reported that HMGA1b is able to enhance the binding affinity of the NF-κB complexes to specific promoters [25], we then asked whether the protein complex CBX7/HMGA1b might involve also NF-κB in the regulation of the *SPP1* gene expression. To test this hypothesis, we first evaluated the effect of CBX7 and HMGA1b on the activity of NF-κB. TPC-1 and B-CPAP cells, showing high and low activity of NF-κB, respectively [26] (Supplementary Figure 3A), were co-transfected with a vector encoding the *luciferase* gene under the control of a NF-κB-responsive promoter (Ig-κB-luc [27]) and increasing amount of a vector expressing CBX7-HA or HMGA1b-HA. By this approach we found that CBX7 was able to reduce the activity of the NF-κB complex, while HMGA1b induced it in a dose-dependent manner (Supplementary Figure 3B and 3C). Therefore, we evaluated by luciferase assays the effects of NF-κB on the modulation of the *SPP1* promoter activity in presence of CBX7 or HMGA1b protein and a FLAG-tagged IκBαM (IκBαM-FLAG) protein, a dominant negative form of the NF-κB-inhibitor IκBα that has been mutated in order to render it unresponsive to the phosphorylation mediated by the IKK proteins that, consequently, are not able to activate NF-κB [27]. TPC-1 cells expressing CBX7 or IκBαM showed a reduced activation of the *SPP1* promoter activity in comparison with cells transfected with the only empty vector, and this reduction was more pronounced when both proteins were expressed (Figure 5A, left panel). In addition, B-CPAP cells transfected with the vector encoding HMGA1b confirmed the positive regulation of the *SPP1* promoter while IκBαM was able to block it (Figure 5A, right panel). Conversely, when both HMGA1b and IκBαM were expressed, we obtained a low activity of the *SPP1* promoter comparable to those observed in presence of the only IκBαM (Figure 5A, right panel). These results were then confirmed by analyzing the *SPP1* expression levels by qRT-PCR assays performed in TPC-1 and B-CPAP cells expressing IκBαM-FLAG and CBX7-HA or HMGA1b-HA, respectively. Consistently with the data obtained by luciferase assays, we found that both CBX7 and IκBαM were able to reduce the expression levels of the *SPP1* gene in TPC-1 cells (Figure 5B), whereas HMGA1b and IκBαM exerted an opposite transcriptional effect on the *SPP1* gene in B-CPAP cells (Figure 5C), in fact, when these latter were both expressed, HMGA1b was not able anymore to exert its role of positive regulator of the *SPP1* expression (Figure 5C). Taken together, these data demonstrate that CBX7 and HMGA1b are able to act in opposite way in the *SPP1* regulatory mechanism that also involves the NF-kB complex.

## DISCUSSION

CBX7 is a Polycomb protein that positively or negatively modulates the expression of several genes involved in development and differentiation by recognizing and binding specific histone modification sites on the chromatin structure [1–4]. The role that CBX7 exerts in cancer is still controversial and not defined at all. In fact, it has been demonstrated that CBX7 acts both as an oncogene and a tumor suppressor gene depending on the cellular system in which it is expressed and on the different proteins interacting with it in the several tissues [28, 29]. Indeed, even if it is able to contribute to the development of T and B cell lymphomas in mouse models [8] and to the expansion of cellular lifespan in human prostate primary epithelial cells and MEFs [6, 28], nevertheless CBX7 plays a key role to prevent carcinogenesis in several systems [9–14]. In addition, our recent study has demonstrated that one of the genes negatively regulated during thyroid carcinogenesis by CBX7 is *SPP1*, encoding the chemokine osteopontin [19] whose expression resulted increased during tumor progression [20, 22] and strongly associated with advanced tumor stages and poor prognosis [21, 30–32].

In the present study we have characterized the molecular mechanism by which CBX7 is able to negatively regulate the *SPP1* gene expression. In particular, since recently we have demonstrated that CBX7 exerts its role of transcriptional regulator by interacting with the HMGA1b protein [13], we evaluated whether the CBX7/HMGA1b complex might be involved in the regulation of the *SPP1* expression.

By evaluating the transcriptional effect of HMGA1b on the *SPP1* gene expression, we found that HMGA1b is able to induce the activity of the *SPP1* promoter, thus positively regulating its expression. This positive regulation was also confirmed by observing a strong reduction of the *Spp1* expression in MEFs obtained from *Hmga1^−/−^* mice compared with that observed in wild type MEFs. Therefore, in this system, HMGA1b seems to act in contrast with the effect of CBX7 on the *SPP1* activation.

Interestingly, we found that CBX7 and HMGA1b counteract each other on the *SPP1* promoter, as evaluated *in vivo* by ChIP assays. Then, by luciferase and qRT-PCR assays we found that when the two proteins were expressed both the *SPP1* promoter activity and the *SPP1* expression decreased, showing levels comparable to those observed in presence of the only CBX7 protein. These results indicate that the transcriptional effect of CBX7 on *SPP1* is stronger than the HMGA1b one and suggest that the loss of CBX7 during cancer progression could trigger the onset of a fully malignant phenotype by allowing HMGA1b to exert its transcriptional activity.

In addition, this transcriptional regulation is reflected in the modulation of cell migration, that is one of the malignancy-related effect of the osteopontin [30]. In fact, while HMGA1b is able to promote cell migration by enhancing the *SPP1* transcription, CBX7 reduces cell motility through the down-regulation of the *SPP1* expression.

Finally, since it has been demonstrated that the NF-κB complex is a positive regulator of *SPP1* [23] and that its subunits interact with HMGA1b [25], we asked whether the CBX7/HMGA1b complex might also involve NF-κB in this *SPP1* regulation mechanism. Luciferase and qRT-PCR assays showed that NF-κB is involved in the regulation of the *SPP1* expression, in fact, the inhibition of NF-κB had the same transcriptional effect of CBX7, showing that CBX7 and NF-κB act in opposite way in the regulation of the *SPP1* expression. More intriguingly, we found that when NF-κB is not active, HMGA1b is not able anymore to induce the expression of *SPP1*. Therefore, we suppose that CBX7/HMGA1b/NF-kB could take part in an interesting molecular mechanism that fine modulates the expression of the *SPP1* gene.

In conclusion, our data demonstrate that the loss of CBX7 associated to the increase of HMGA1b during carcinogenesis would contribute to cancer progression through the transcriptional deregulation of the *SPP1* expression.

## MATERIALS AND METHODS

### Cell culture and transfection

HEK 293, TPC-1 and B-CPAP cell lines were grown in DMEM (Life Technologies, Grand Island, NY) supplemented with 10% fetal bovine serum, 1% L-glutamine 10 mM, 1% penicillin/streptomycin (Life Technologies). Mouse embryonic fibroblasts (MEFs) from *Hmga1*-knock-out mice were established as described elsewhere [33] and grown in DMEM supplemented with 1% L-glutamine, 1% penicillin/streptomycin and 1% gentamicin (Life Technologies). All cell lines were maintained at 37°C under 5% CO_2_ atmosphere.

B-CPAP cells were transfected by using Fugene HD reagent (Promega, Fitchburg, WI) while for TPC-1 and HEK 293 cells it was used the Neon Electroporation System (Life Techologies), according to manifacturer's instructions. The expression vectors encoding the CBX7 protein fused to V5 or HA epitope and the vector expressing the HA-tagged HMGA1b protein have been previously described [14, 34]. The pcDNA3.1/IκBαM-FLAG vector, encoding a mutant form (S32A/S36A) of the IκBα protein, was a gift of Prof. Antonio Leonardi from University of Naples “Federico II” [27]. For migration assays, TPC-1 cells were transfected with a short interfering RNA (siRNA) specific for the human *SPP1* mRNA (Flexi Tube Gene Solution, GS6696, Qiagen, Hilden, Germany) or with a non-specific control siRNA (All Stars Negative Control siRNA AF488, 1027292, Qiagen) at a final concentration of 120 nM. For all transfections, the total amount of the transfected DNA was balanced with the empty vector.

### Luciferase assays

2 × 10^5^ TPC-1 and B-CPAP cells were seeded in 24-well plate and transfected with 200 ng of the *SPP1-*luciferase reporter gene or with 100 ng of the Ig-κB-luciferase reporter gene (kindly provided by Prof. Antonio Leonardi [27]). 48 h or 24 h after transfection, respectively, cell extracts were prepared and the luciferase activity was measured by using a Lumat LB9507 luminometer (Berthold Technologies, Bad Wildbad, Germany) and the Dual-Luciferase Reporter System kit (Promega). A vector expressing *Renilla* gene under the control of the cytomegalovirus (CMV) promoter was used to normalize transfection efficiency. For all transfections, the total amount of the transfected DNA was balanced with the empty vector.

### RNA extraction and quantitative (q)RT-PCR

Total RNA was extracted from cell lines and tissues by using Trizol reagent (Life Technologies), according to manifacturer's instructions. 1 μg of total RNA was used to obtain the cDNA with the QuantiTect Reverse Transcription Kit (Qiagen). qRT-PCR analysis was carried out in 96-well plates with the CFX 96 thermocycler (Bio-Rad, Hercules, CA) by using 20 ng of each cDNA and Sybr Green (Applied Biosystems, Foster City, CA) or Real Master Mix (5Prime Inc., Gaithersburg, MD). For human SPP1, CBX7 and GAPDH amplification we used TaqMan gene expression assays (Applied Biosystems, CBX7: Hs00545603_m1; SPP1: Hs00959010_m1; GAPDH: Hs02758991_g1). For human HMGA1b and G6PD we used the following primers:

*HMGA1b Forward* CAACTCCAGGAAGG AAACC,
*HMGA1b Reverse* AGGACTCCTGCGAGATGC,
*G6PD Forward* ACAGAGTGAGCCCTTCTTCAA,
*G6PD Reverse* ATAGGAGTTGCGGGCAAAG.

For mouse Spp1 and G6pd we used the following primers:

*Spp1 Forward* GTGGCCCATGAGGCTGCAGT,
*Spp1 Reverse* GCCAGAATCAGTCACTTTC ACCGGG,
*G6pd Forward* CAGCGGCAACTAAACTCAGA
*G6pd Reverse* TTCCCTCAGGATCCCACAC.

Relative Expression was calculated according to the 2^−ΔΔCt^ formula as previously described [35], and expression value of controls was set equal to 1.

### Protein extraction and Western blot analysis

Total protein extracts were obtained by using the JS lysis buffer (20 mM Tris-HCl pH 7.5, 5 mM EDTA, 150 mM NaCl, 1% Nonidet P40) completed with a mix of inhibitors of proteases and phosphatases. The extracted proteins were separated by SDS-PAGE and then transferred onto Protran membranes (Perkin Elmer, Boston, MA). Membranes were blocked with BSA or 5% non-fat milk and then incubated with the following antibodies: anti-V5 (Invitrogen, Carlsband, CA), anti-HA (Roche Applied Science, Mannheim, Germany), anti-CBX7 (Santa Cruz Biotechnology, Inc., Santa Cruz, CA), anti-FLAG (clone M2, Sigma-Aldrich, St. Louis, MO), anti-osteopontin (ab8448, Abcam, Cambridge, UK). To normalize the amount of protein loaded membranes were incubated with anti-γ-Tubulin, anti-β-Actin and anti-Vinculin (Santa Cruz Biotechnology, Inc.). Membranes were then incubated with horseradish peroxidase-conjugated secondary antibody (1:3000) for 60 min at room temperature and the signals were detected by enhanced chemiluminescence (ECL) detection system (Thermo Fisher Scientific, Inc., Waltham, MA). Human osteopontin recombinant full length protein was purchased from Life Technologies.

### Chromatin immunoprecipitation (ChIP) assays

ChIP experiments were performed as reported elsewhere [14]. Briefly, 48 h after transfection, 5 × 10^6^ HEK 293 cells were cross-linked to fix the DNA-protein complexes using 1% formaldehyde at room temperature for 10 min and the reaction was then stopped by adding glycine at a final concentration of 0.125 M. Cells were lysed in 300 μl of buffer containing 10 mM EDTA, 50 mM Tris-HCl pH 8.0, 1% SDS and protease inhibitors and then sonicated three times for 10 minutes at maximum settings (Bioruptor^TM^ Next Gen, Diagenode Inc., Denville, NJ), obtaining fragments between 0.3 and 1.0 kb. After centrifuging samples at 14000 rpm for 15 minutes at 4°C, 6% of supernatant amount was used as control of the total chromatin obtained (input), and the remaining part of the sample was diluted 2.5-fold in IP buffer (100 mM NaCl, 2 mM EDTA pH 8.0, 20 mM Tris-HCl pH 8.0, 0.5% Triton X-100 and protease inhibitors). After 3 h of pre-clearing at 4°C with protein A- or protein G-Sepharose saturated with salmon sperm (Millipore, Billerica, MA), samples were mixed overnight at 4°C with the following antibodies: anti-HA (Santa Cruz Biotechnology, Inc.), anti-V5 (Sigma), aspecific IgG (Santa Cruz Biotechnology, Inc.). Subsequently, the DNA-protein-antibodies complexes were immunoprecipitated with the proteins A/G previously used and then the chromatin was released from the beads through 30 minutes incubation with 250 μl of 1% SDS, 0.1 M NaHCO_3_ at 37°C and finally with 200 nM NaCl at 65°C overnight. Subsequently, 10 μl of 0.5 mM EDTA, 20 μl of 1 M Tris-HCl pH 6.5 and 20 μg of Proteinase K were added to the reaction tube and then the complexes were incubated for 1 h at 45°C. DNA from chromatin immunoprecipitated was purified by phenol/chloroform extraction (Life Technologies) and precipitated by adding two volumes of ethanol and 0.1 M CH_3_COONa. Finally, 20 ng of chromatin were analyzed by qRT-PCR assays using the following primers:

*promoter SPP1 Forward*: CATTAATGTTTTTCCC TACTTTCTCC,
*promoter SPP1 Reverse*: TCATTAACTAGCTTTTT CATTTACGG,
*promoter GAPDH Forward*: CCCAAAGTCCTCC TGTTTCA,
*promoter GAPDH Reverse: GTCTTGAGGCCTG AGCTACG*.

The percentage of IP chromatin was calculated as 2^−ΔCt^ × 6, where ΔCt is the difference between Ct_input_ and Ct_IPsample_, and 6 is the percentage of total sample used for the input (see above). All ChIP data were from at least two independent experiments, and for each experiment qRT-PCR assay was performed at least in duplicate.

### Cell migration assays

Transwell motility assays were performed using 8 micron pore, 6.5 mm polycarbonate transwell filters (Corning Costar Corp., Cambridge, MA). 24 h after transfection, 2.5 × 10^4^ TPC-1 cells were suspended in 200 μl serum-free medium and seeded on the upper surface of the filters and allowed to migrate toward 300 μl of 10% FBS-containing medium in the bottom compartment. Moreover, 2.5 × 10^4^ cells were plated in a 96-well plate in triplicate and treated with CellTiter96 Aqueous One (Promega) to confirm that we used the same number of cells for each condition. After additional 24 h, transwell filters were washed three times with PBS at room temperature and cells migrated to the underside of filters were fixed and stained with crystal violet solution (0.1% crystal violet, 20% methanol). Cells remaining on the upper surface were removed with a cotton swab. In order to exactly quantify crystal violet staining samples were de-stained with 1% SDS in 300 μl of PBS, the absorbance of eluates were read at 595 nm in microplate reader (LX 800, Universal Microplate Reader, BioTek, Winooski, VT) and then normalized with CellTiter values. Extracted RNA and proteins were analyzed to evaluate the expression of the transfected vectors.

### Statistical analysis

All data were tested for normality distribution using Shapiro-Wilk test for all variables. Nonparametric Mann Whitney test and nonparametric Kruskal-Wallis test were used to evaluate the statistical significance of the obtained data. When Kruskal-Wallis test was significant (*p* < 0.05), we determined the differences between groups using Dunn's post test. In all the experiments, the significance was considered for *p* < 0.05. Data are reported as mean values ± standard error of mean (SEM).

## SUPPLEMENTARY FIGURES


