# Supplementary material for: CBX7 and HMGA1b proteins act in opposite way on the regulation of the SPP1 gene expression
Source: Oncotarget. 2015 Jan 20;6(5):2680–92. doi: 10.18632/oncotarget.2777 (PMC4413610; doi:10.18632/oncotarget.2777)
Supplement: Supplementary file 1 [file oncotarget-06-2680-s001.pdf]

SUPPLEMENTARY FIGURES

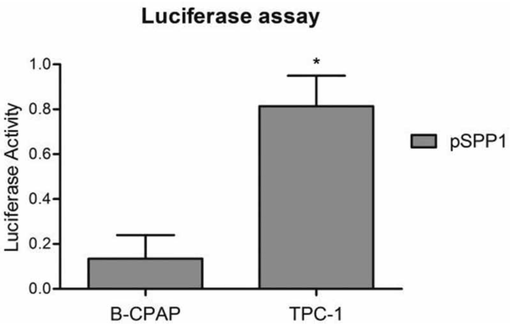

Supplementary Figure 1: *SPP1* promoter activity in B-CPAP and TPC-1 cell lines. Luciferase assays were performed in B-CPAP and TPC-1 cells transfected with the *SPP1-luc* promoter. Values are the mean of four (B-CPAP) or five (TPC-1) independent experiments performed at least in duplicate ± SEM. Mann Whitney test was used to correlate the different endogenous activity of the *SPP1* promoter in the two different cell lines (*$p < 0.05$).

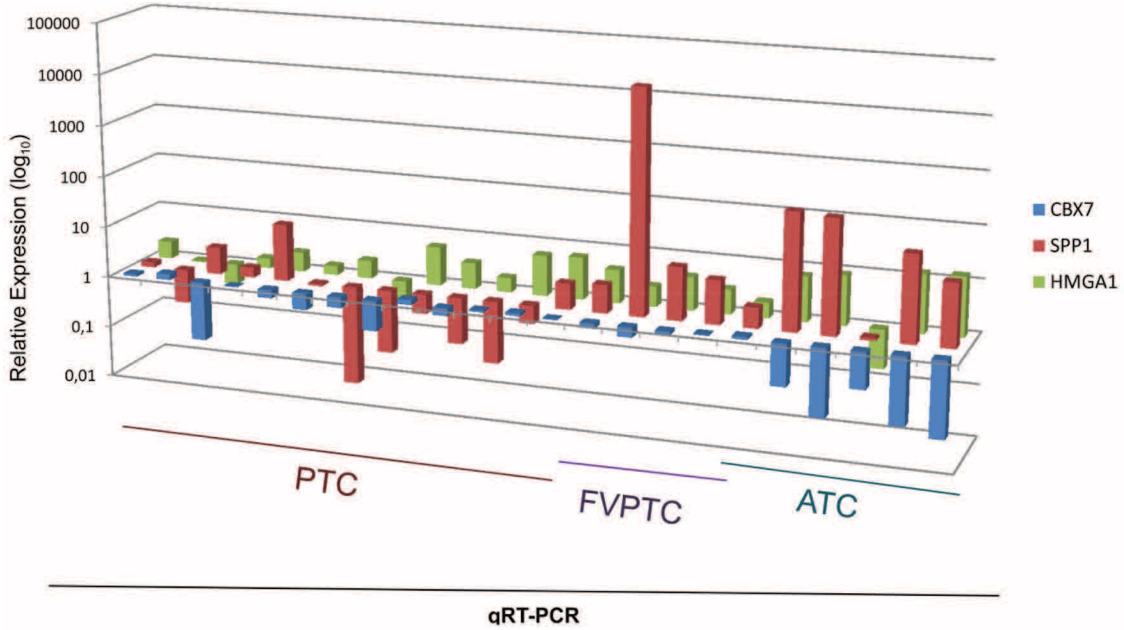

Supplementary Figure 2: Expression analysis of *CBX7*, *HMGA1b* and *SPP1* in a panel of human thyroid carcinomas. qRT-PCR assay was performed in 14 papillary thyroid carcinomas (PTC), 4 follicular variant papillary carcinomas (FV-PTC) and 5 anaplastic thyroid carcinomas (ATC), obtained from the Centre Hospitalier Lyon Sud, Pierre Bénite, France. Relative Expression represents gene expression values of each sample normalized to those observed in the mean of three normal thyroid samples, set equal to 1. qRT-PCR assay was performed in duplicate.

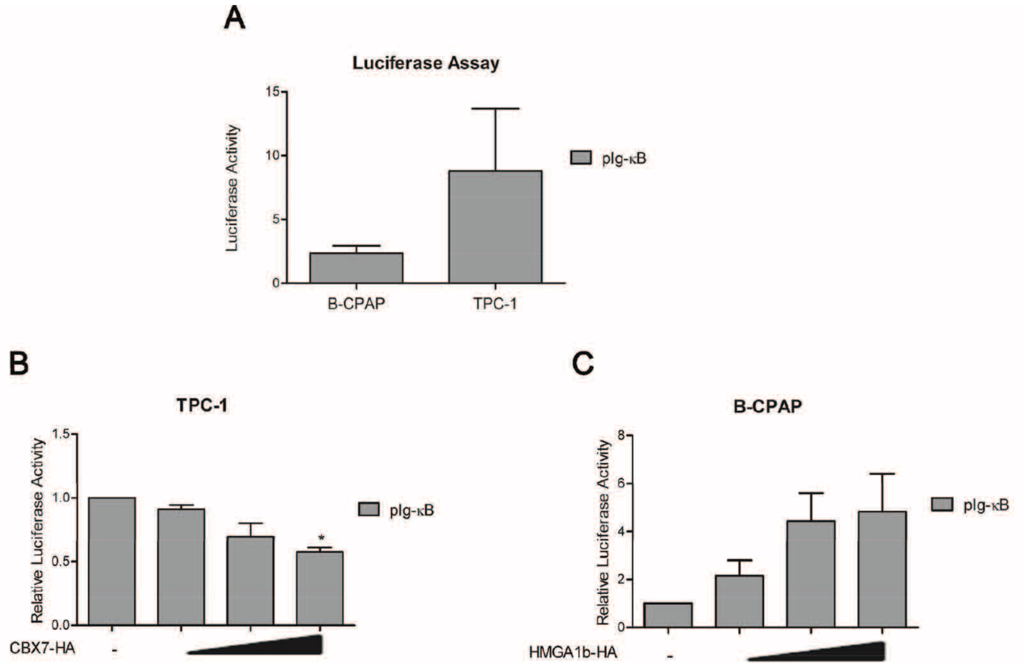

Supplementary Figure 3: CBX7 and HMGA1b modulate the activity of NF-κB. **(A)** Luciferase assays were performed in B-CPAP and TPC-1 cells to evaluate the endogenous activity of NF-κB. Represented values are the mean of three independent experiments performed at least in duplicate ± SEM. **(B)** Luciferase assays performed in TPC-1 cells transfected with the *Ig-κB-luc* vector and with increasing amount of a vector expressing CBX7-HA. The total amount of the transfected DNA was balanced with the empty vector. Relative Luciferase Activity (pIg-κB) was normalized to that observed in cells transfected with the only empty vector, set equal to 1. Values are the mean of three independent experiments performed in triplicate ± SEM. Kruskal-Wallis test followed by Dunn's post test: $^*p < 0.05$ compared to cells transfected with the only empty vector. **(C)** Luciferase assays performed in B-CPAP cells transfected with the *Ig-κB-luc* vector and increasing amount of a vector expressing HMGA1b-HA. The total amount of the transfected DNA was balanced with the empty vector. Relative Luciferase Activity (pIg-κB) was normalized to that observed in cells transfected with the only empty vector, set equal to 1. Values are the mean of four independent experiments performed in triplicate ± SEM.
